# Clinical validity of outcome pain measures in naturally occurring canine osteoarthritis

**DOI:** 10.1186/1746-6148-8-162

**Published:** 2012-09-10

**Authors:** Pascale Rialland, Sylvain Bichot, Maxim Moreau, Martin Guillot, Bertrand Lussier, Dominique Gauvin, Johanne Martel-Pelletier, Jean-Pierre Pelletier, Eric Troncy

**Affiliations:** 1 Department of Biomedical Sciences, GREPAQ (Research Group in Animal Pharmacology of Quebec), St.-Hyacinthe (QC), J2S 7C6, Canada; 2 Osteoarthritis Research Unit, University of Montreal Hospital Research Center (CRCHUM), Notre-Dame Hospital, Montreal (QC), H2L 4 M1, Canada; 3 The Companion Animal Research Group; Faculty of Veterinary Medicine, Université de Montréal, St.-Hyacinthe (QC), J2S 7C6, Canada

**Keywords:** Psychometrics, Dog osteoarthritis, Pain, Metrology, Kinetics, Accelerometry, Behavioral scales, Skin conductance

## Abstract

**Background:**

The conceptual validity of kinetic gait analysis and disability outcome assessment methods has guided their use in the assessment of pain caused by osteoarthritis (OA). No consensus on the best clinical methods for pain evaluation in canine OA exists, particularly, when evaluating treatments where a smaller treatment effect is anticipated than with pharmacological pain killers. This study thus aimed at determining the technical validity of some clinical endpoints on OA pain in dogs using the green-lipped mussel (GLM)-enriched diet.

Twenty-three adult dogs with clinical OA completed the prospective controlled study. All the dogs were fed a balanced diet over a 30-day control period followed by a GLM-enriched diet over a 60-day period. The kinetic gait analysis parameter (PVF_BW_, peak vertical force adjusted for body weight change), electrodermal activity (EDA), and a standardized multifactorial pain questionnaire (MFQ) were performed on day (D) 0 (inclusion), D30 (start) and D90 (end). The owners completed a client-specific outcome measures (CSOM) instrument twice a week. Motor activity (MA) was continuously recorded in seven dogs using telemetered accelerometric counts. We hypothesized that these methods would produce convergent results related to diet changes. A Type I error of 0.05 was adjusted to correct for the multiplicity of the primary clinical endpoints.

**Results:**

Neither the EDA nor the MFQ were found reliable or could be validated. Changes in the PVF_BW_ (P_adj_ = 0.0004), the CSOM (P_adj_ = 0.006) and the MA intensity (P_adj_ = 0.02) from D0 to D90 suggested an effect of diet(s). Only the PVF_BW_ clearly increased after the GLM-diet (P_adj_ = 0.003). The CSOM exhibited a negative relationship with the PVF_BW_ (P = 0.02) and MA duration (P = 0.02).

**Conclusions:**

The PVF_BW_ exhibited the best technical validity for the characterization of the beneficial effect of a GLM-enriched diet. The CSOM and MA appeared less responsive following a GLM-diet, but these measures appeared complementary to gait analysis. Apparently, the CSOM provides the capacity to rely on pain OA assessment influenced by both lameness quantification (PVF_BW_) and physical functioning (MA).

## Background

The prevalence of osteoarthritis (OA) in the canine population (20% of adult and 80% of the geriatric (> 8 years old) dogs in North America [1]) makes the disease a major cause of concern. The distortion between clinical and radiographic finding in dog OA is well recognized [2]. The symptomatic signs of OA are highly variable, related to pain and physical functioning, and translated into limb impairment, activities limitations and restricted participation [3-5]. This situation led to the use of multiple methods to assess the efficacy of OA treatment [6]. However, interpretations are not always clear, the results are often inconsistent and clinical validation of these methods is often missing.

The conceptual validity^a^ of the canine pain scales designed for OA and chronic pain has evolved over time [7-11]. These scales have demonstrated their sensitivity to detect response to OA treatment where pain was reduced following nonsteroidal anti-inflammatory drug (NSAID) [11-13], nutraceutical [14] or other nontraditional [13,15] treatment for OA. However, divergent interpretations were addressed when comparing subjective pain scores to an objective evaluation of lameness [13,14], emphasizing the limited information on the relationship between pain scales and OA severity. The majority of standardized multifactorial pain questionnaires (MFQ) are guided by the traditional notion of an inevitably progressive and degenerative disease process; greater behavioral changes indicate higher OA severity and pain in dogs. However, OA is a heterogeneous group of painful conditions [16,17]. The variety of OA signs suggests the need for the implementation of a clinical assessment that includes a broader variable profile than the current standardized OA profile. For this purpose, the client-specific outcome measures (CSOM) require the owner to report pain behavioral changes and/or impaired activities in their OA-afflicted dogs [18,19]. However, the promising CSOM have not met the technical validity^b^ criteria [19]. These data suggest that psychometric^c^ support for the CSOM instrument is required.

The enhancement of a measurement’s interpretability requires the referencing of its value to another measurement with established conceptual validity and interpretability. A surrogate to pain evaluation, vertical ground reaction forces, such as PVF and vertical impulse, measured through objective kinetic analysis have prevailed over pain measurements to quantify limb impairment [20,21]. Interestingly, the PVF variable showed sensitivity^d^ and responsiveness^e^ for the efficacy of the anti-inflammatory, analgesic and structural effects of NSAID [22-25] and nontraditional [13] treatments, which supports a regulatory claim for these treatments for osteoarthritic dogs.

Physical activity represents a distinct dimension of physical functioning [26], and as such, telemetered motor activity (MA) is an objective behavioral method for the clinical assessment of physical function and overall well-being for canine OA [27,28]. However, more data are required to confirm its validity for pain evaluation in OA clinical trials.

Clinicians must be able to interpret the literature to implement the best evidence-based practices. Therefore, here we performed a preliminary analysis to determine the reliability^f^, responsiveness and criterion validities^g^ for canine pain assessment in a one-way crossover clinical trial in naturally occurring OA in dogs that were fed with two successive diets (control and therapeutic) during a 90-day period. We further hypothesized, first, that changes in measurements of the clinical endpoints (dependent variables) would arise from the diets (independent variable) with every other possible factor controlled and, second, that validated outcomes would correlate strongly to PVF as the primary outcome and MA as an exploratory outcome. This study determined the clinical utility of selected methods regardless of some technical validities.

## Results

### Animals

A total of 30 dogs were initially recruited. Seven dogs were excluded for the following reasons: three due to a sudden deterioration of their condition (one dog at D21 and two dogs at D45 and D52), three for a lack of owner follow-up, and one for NSAID use (at D40). The sample included 14 males and 9 females, and the most frequent breeds were Labrador (n = 7) and Golden Retrievers (n = 4). The remainder of the dogs were mixed-breeds (n = 3) and various pure-breeds (n = 9). The dogs were 2.5 to 11.5 years old (6 years, median) at the time of inclusion and weighed 25.2 to 68.4 kg [40.4 ± 1.8 kg]. The joint that caused the most lameness in each animal during the orthopedic examination was the hip (34.8%) followed by the stifle (30.4%), shoulder and elbow (13.0%, each), and carpus and tarsus (4.4% each). Joint pathologies included hip dysplasia (39.1%), cranial cruciate ligament rupture (26.1%), elbow dysplasia (13.1%), shoulder osteochondritis dissecans (OCD) (13.0%), tarsus OCD, and carpus OA with no obvious origin (4.4% each).

### Primary endpoints

#### Kinetic force platform gait analysis (PVF_BW_)

**Figure 1:**
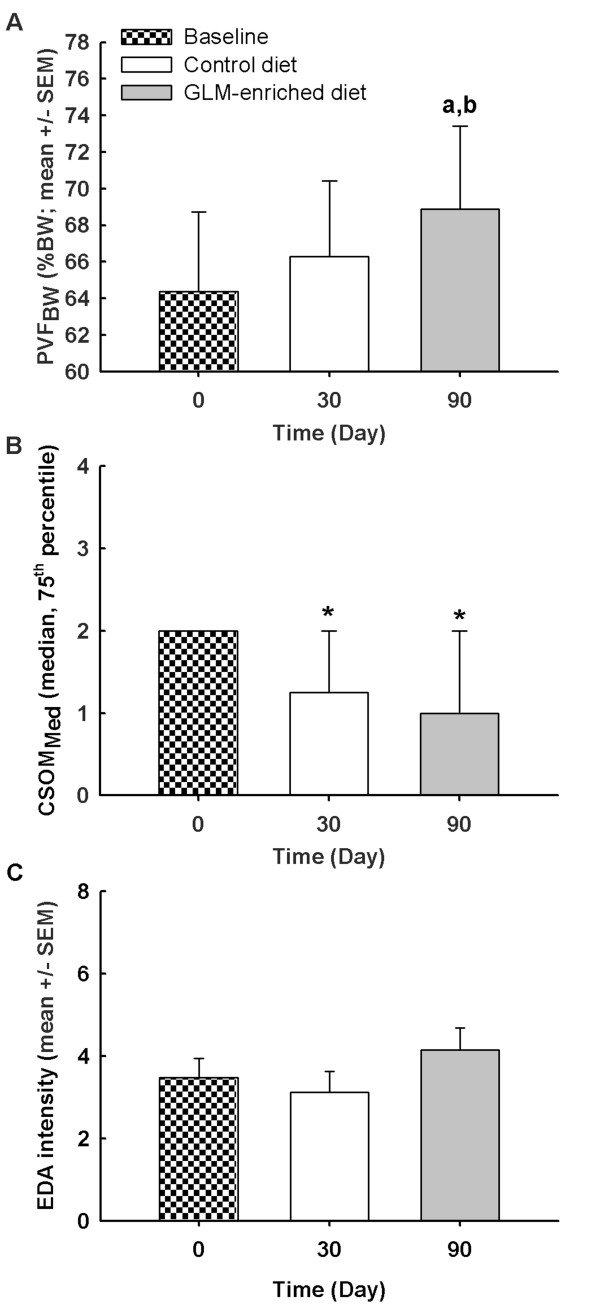
**Evolution of primary clinical endpoints in osteoarthritic dogs fed subsequentially with control and green-lipped mussel-enriched diets.A**) PVF_BW_: Peak vertical ground reaction force adjusted to change in body weight (%BW). **B**) CSOM_*Med*_: Box plot of the median-value of client-specific outcome measures (score). **C**) EDA: electrodermal activity. Data are represented as mean ± SEM for PVF_BW_ and EDA, and as median + 75^th^ percentile for CSOM_*Med*_ at day(D)0, D30, and D90. General linear mixed model for repeated measures: (**a**) and (**b**) were significant difference of Least Squares Means when compared with D0 and D30, respectively. Multinomial logistic regression for repeated measures: *significant odds ratio (when compared to D0). Significance was reached at P-value lower than 0.017 with Bonferroni’s adjustment.

The mean CV for PVF_BW_ was not different over time (P = 0.82) (Table 1). A significant effect of the anatomical location of the most affected limb on the PVF_BW_ was observed (P < 0.001). Moreover, the PVF_BW_ was significantly higher on D90 than on D0 (P_adj_ = 0.0004) and D30 (P_adj_ = 0.003) (Figure 1A). No differences between D0 and D30 were observed (P_adj_ = 0.06).

**Table 1:** **Mean dog coefficient of variation**^**a**^**on the primary clinical endpoints**^**b**^

**Variable**	**D0 mean (SD)**	**D30 mean (SD)**	**D90 mean (SD)**	**Time effect**^**c**^**F value (P)**
PVF_BW_	3.9 (2.2)	3.6 (1.8)	4.0 (3.2)	0.19 (0.82)
CSOM	11.3 (16.3)	5.7 (12.1)	3.9 (10.5)	0.94 (0.34)
EDA	15.6 (14.4)	11.4 (12.7)	10.7 (8.6)	**16.76 (< 0.001)**

^a^Coefficient of variation (CV) is calculated as the ratio of the standard deviation to the mean. ^**b**^*PVF*_*BW*_: peak vertical ground reaction force adjusted for body weight change; *CSOM*: client-specific outcome measures; *EDA*: electrodermal activity.

Descriptive data are represented as mean (SD).^c^The mean CV were considered not variable at each time point, when time did not exert a significant effect with P-value higher than 0.05.

##### Client-specific outcome measures (CSOM)

The mean CV for CSOM was not different over time (P = 0.34) (Table 1). The CSOM_*Med*_ was higher on D0 than on D30 [odds ratio (OR): 3.8, 95% confidence interval (CI): 1.4 to 9.8, P_adj_ = 0.03] and on D90 [OR: 6.7, CI: 2.0 – 22.6, P_adj_ = 0.006] in the dogs with OA (Figure 1B). No difference between D30 and D90 was noted [OR: 1.8, CI: 0.7 – 5.0, P_adj_ = 1.00]. The ranked activities (Table 2) revealed that the second ranked activity (Act_(2)_) was significantly different between D0 and D30 [OR: 3.5, CI:1.3 – 9.2, P_adj_ = 0.04] and D90 [OR: 8.7, CI: 2.2 – 34.1, P_adj_ = 0.006]. No differences over time were observed for the other ranked activities, which suggested that the ranking of the activities had no/poor influence.

**Table 2:** Client-specific outcome measures (CSOM)

	**Time**	**Median (range)**	**Statistical comparison**	**OR (95% LCL – UCL)**	**Chi square**	**P**
Act_(1)_	D0	2 (0 – 4)	D0 *vs*. D30	2.6(1.0 – 6.9)	4.9	0.04
	D30	1 (1 – 3)	D0 *vs*. D90	5.3(1.3 – 21.3)	5.5	0.019
	D90	1 (0 – 3)	D30 *vs*. D90	1.9(0.5 – 7.1)	1.1	0.29
Act_(2)_	D0	2 (1 – 4)	D0 *vs*. D30	3.5*(1.3 – 9.2)	6.4	**0.011**
	D30	2 (0 – 4)	D0 *vs*. D90	8.7*(2.2 – 34.1)	9.7	**0.0018**
	D90	1 (0 – 2)	D30 *vs*. D90	2.5(0.8 – 6.9)	3	0.08
Act_(3)_	D0	2 (1 – 3)	D0 *vs*. D30	1.8(0.8 – 4.2)	2.0	0.15
	D30	2 (0 – 3)	D0 *vs*. D90	2.8(0.9 – 8.4)	3.3	0.06
	D90	1 (0 – 4)	D30 *vs*. D90	1.5(0.4 – 5.0)	0.5	0.47
Act_(4)_	D0	2 (1 – 4)	D0 *vs*. D30	2.0(0.8 – 4.7)	2.5	0.10
	D30	2 (0 – 4)	D0 *vs*. D90	4.2(1.1 – 16.2)	4.4	0.03
	D90	2 (0 – 4)	D30 *vs*. D90	2.1(0.7 – 5.9)	2.0	0.15
Act_(5)_	D0	2 (1 – 3)	D0 *vs*. D30	_	9.1	0.02
CMH	D30	2 (0 – 3)	D0 *vs*. D90	_	7.5	0.05
	D90	1 (0 – 3)	D30 *vs*. D90	_	3.5	0.17
Ctg_(1)_	D0	2 (1 – 4)	D0 *vs*. D30	2.7*(1.4 – 5.3)	8.3	**0.004**
	D30	2 (0 – 2)	D0 *vs*. D90	2.6(1.7 – 6.6)	4.6	0.03
	D90	1 (0 – 3)	D30 *vs*. D90	0.9(0.4 – 2.1)	0.4	0.97
Ctg_(2)_	D0	2.5 (0 – 3)	D0 *vs*. D30	10.5*(1.5 – 72.0)	5.7	**0.016**
	D30	2 (0 – 2)	D0 *vs*. D90	32.7*(2.3 – 457.0)	6.7	**0.009**
	D90	1 (0 – 2)	D30 *vs*. D90	3.1(0.7 – 13.3)	2.3	0.12
Ctg_(3)_	D0	2 (1 – 4)	D0 *vs*. D30	0.8(0.3 – 2.3)	0.1	0.73
	D30	2 (1 – 4)	D0 *vs*. D90	0.6(0.2 – 1.6)	0.8	0.35
	D90	2 (0 – 3)	D30 *vs*. D90	0.2(0.2 – 4.7)	0.09	0.76
Ctg_(4)_	D0	2 (2 – 2)	D0 *vs.* D30	_	4.1	0.25
CMH	D30	2 (1 – 2)	D0 *vs.* D90	_	13.0^♦^	**0.011**
	D90	1 (0 – 2)	D30 *vs*. D90	_	6.6	0.08
Ctg_(5)_	D0	2 (2 – 3)	D0 *vs*. D30	_	0.11	0.73
CMH	D30	1.5 (1 – 2)	D0 *vs*. D90	_	0.55	0.45
	D90	1 (1 – 2)	D30 *vs*. D90	_	0.11	0.73

Selected activities, Act_(1)_ to Act_(5)_ ranked by decrescendo order of importance by owner. Selected behavioural categories, *Ctg*_*(1)*_: Reduced mobility; *Ctg*_*(2)*_: Reduced mobility after exercise; *Ctg*_*(3)*_: Reduced ability to change posture; *Ctg*_*(4)*_: Reduced ability to change posture after rest or in the morning; *Ctg*_*(5)*_: Resistance to manipulations. *LCL*: lower control limit; *UCL*: upper control limit.

Multinomial logistic regression for repeated measures: *significant odds ratio *(OR)*. Cochran-Mantel-Haenszel *(CMH)* test: ^♦^significant row mean scores difference *(RMSD)*. Estimates were significant P-value (P) lower than 0.017 with Bonferroni’s adjustment.

The selected activities were categorized as follows: 41 activities were Ctg_(1)_ (reduced mobility), 16 activities were Ctg_(2)_ (reduced mobility after exercise), 12 activities were Ctg_(3)_ (reduced ability to change posture), 10 activities were Ctg_(4)_ (reduced ability to change posture after rest or in the morning), 4 activities were Ctg_(5)_ (resistance to manipulations), and one activity was Ctg_(6)_ (mood change). The Ctg_(1)_ was higher on D0 than on D30 [OR: 2.7, CI: 1.4 – 5.3, P_adj_ = 0.02], but no difference was found between the other time points (Table 2). The Ctg_(2)_ was higher on D0 than on D30 [OR: 10.5, CI: 1.5 – 72.0, P_adj_ = 0.05] and on D90 [OR: 32.7, CI: 2.3 – 457.0, P_adj_ = 0.03] (Table 2). The Ctg_(4)_ revealed a significantly higher score on D0 than on D90 (Row Mean Score Difference, RMSD = 13.0, df = 1, P_adj_ = 0.04) (Table 2). No differences over time were observed for Ctg_(3)_ and Ctg_(5)_ (Table 2).

#### Electrodermal activity (EDA)

The mean CV for EDA was different over time (P < 0.001) (Table 1), which suggested EDA was variable on repeated measurement throughout the study. This poor technical reliability precludes any interest in the use of the measurement for the assessment of treatment responsiveness. The EDA was not different on D0 compared with D30 (P_adj_ = 0.99) and D90 (P_adj_ = 0.90). No difference was observed between D30 and D90 (P_adj_ = 0.07) (Figure 1C).

##### Criterion validities

The pairwise correlation coefficients between the PVF_BW_ and the CSOM_*Med*_ were low (Spearman's rank correlation coefficient (rho_S_) = −0.03, 0.10 and −0.37 on D0, D30 and D90, respectively) (Table 3). Some relationship between the PVF_BW_ and the CSOM_*Med*_ appeared between D30 and D90 using the descriptive pairwise correlation coefficient (rho_S_ = −0.71) (Table 3). The regression estimate between the PVF_BW_ and the CSOM_*Med*_ was significant (estimate (SE) = −0.01 (0.003), P = 0.02). These results indicate that both variables are convergent and that the CSOM_*Med*_ change is predictive, in some part, of the PVF_BW_ change. The CSOM categories were presented as exploratory endpoints because they were *a posteriori* measures with interesting information (Table 3). Descriptive correlations revealed that the CSOM categories reflected the CSOM_*Med*_*.*

**Table 3:** **Spearman's rank correlation coefficient of PVF**_**BW**_**in comparison with measures**^**a**^

**Comparison of PVF**_**BW**_**with:**	**n**	**D0 (95% CI)**	**D30 (95% CI)**	**D90 (95% CI)**	**Difference D0D30 (95% CI)**	**Difference D30D90 (95% CI)**
Primary endpoints						
CSOM	23	-.03 (−.64, .32)	.10 (−.38, .57)	-.37 (−.74, .12)	.13 (−.44, .66)	**-.71 (−.91, -.34)**
EDA	23	-.23 (−.61, .24)	.10 (−.44, .60)	.16 (−.36, .62)	-.12 (−.60, .43)	.31 (−.23, .69)
Exploratory endpoints						
MA	7	-.46 (−1.0, .77)	.10 (−1.0, 1.0)	.45 (−.68, 1.0)	-.04 (−.94, 1.0)	.14 (−1.0, 0.80)
Ctg_(1)_	20	-.20 (−.65, .42)	-.29 (−.66, .21)	-.13 (−.58, .40)	-.03 (−.67, .74)	**-.64 (−.87, -.15)**
Ctg_(2)_	10	.01 (−.77, .78)	.00 (−.77, .79)	**-.75 (−1.0, 0.00)**	-.03 (−.81, .74)	-.52 (−.90, .45)
Ctg_(3)_	9	.56 (−.72, 1.0)	.54 (−.31, 1.0)	.52 (−.81, 1.0)	.00 (−.91, .92)	**-.99 (−1.0, -.87)**
Ctg_(4)_	8	.20 (−.44, .94)	.09 (−.91, 1.0)	.07 (−.91, 1.0)	.20 (−.94, 1.0)	-.66 (−1.0, .71)

*PVF*_*BW*_: peak vertical ground reaction force adjusted for body weight change.

^a^*CSOM*: client-specific outcome measures; *EDA*: electrodermal activity; *MA*: motor activity; *Ctg*_*(1)*_: Reduced mobility; *Ctg*_*(2)*_: Reduced mobility after exercise; *Ctg*_*(3)*_: Reduced ability to change posture; *Ctg*_*(4)*_: Reduced ability to change posture after rest or in the morning.

Descriptive data are represented as Spearman's rank correlation coefficient at day(D)0, D30, D90, difference between D0 and D30 (D0D30), and difference between D30 and D90 (D30D90). 95% CI: 95% confidence interval from bootstrap method of sample of n dogs (1000 samples randomly selected with replacement). NA: not available.

The pairwise correlation coefficient between the PVF_BW_ and the EDA indicated no linear relationship on D0, D30, and D90 or differences between D0 and D30 and D30 and D90 (Table 3). Regression analyses demonstrated no relationship between the PVF_BW_ and the EDA (estimate (SE) = 0.001 (0.001), P = 0.91), which implies that EDA had no empirical association with PVF_BW_.

### Exploratory endpoints

#### Motor activity (MA)

**Figure 2:**
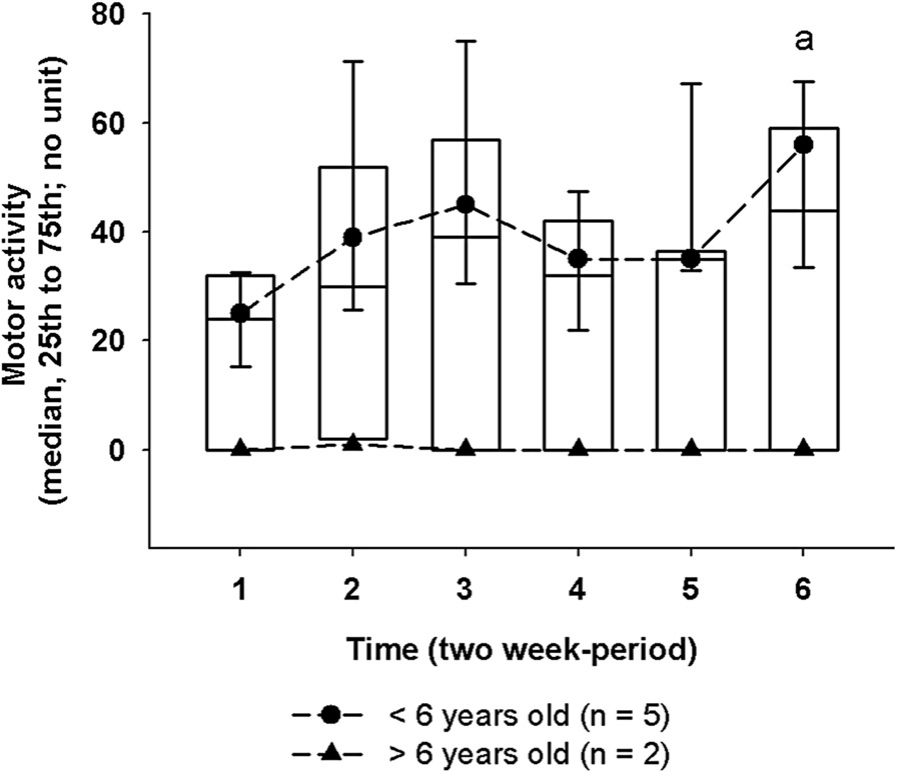
**Evolution of motor activity in osteoarthritic dogs fed subsequentially with control and green-lipped mussel-enriched diets.** Motor activity (MA) is represented as box plot (median and 25^th^ to 75^th^ percentile) per two consecutive weeks (periods P_(i)_) for n = 7 dogs. General linear mixed model for repeated measures: ^a^significant difference of Least Squares Means when compared with P1. Significance was reached at P-value lower than 0.003 with Bonferroni’s adjustment. As descriptive statistics, MA was presented by age category, and represented as median (25^th^ to 75^th^ percentile) for dogs under 6-y (black circle and short dashed lines) and dogs older than 6-y (black triangle and long dashed line).

The mixed model of MA recordings in the seven randomized dogs revealed a significant main effect for time (P = 0.04), age (P < 0.001) and daily period (P < 0.001). A significant effect of age was observed between dogs older than 6 years and younger dogs (minus 6 years) (P < 0.001) (Figure 2). A significant effect of the daily period on MA intensity was noted because the dogs were less active at night than during the morning (P = 0.002) or the afternoon (P < 0.001). The planned comparisons showed that MA intensity was lower at P1 compared to P6 (P_adj_ = 0.02) (Figure 2).

There was no pairwise correlation (Table 3) and no relationship between the MA intensity and the PVF_BW_ (P = 0.36). No relationship between the MA intensity and the CSOM_*Med*_ (P = 0.79) was noted on the regression analysis. However, there was a significant negative relationship between the MA duration and the CSOM_*Med*_ (P = 0.02).

#### Multifactorial pain questionnaire (MFQ)

The internal consistency of the MFQ-S (*n*_*ij*_ = 69, Cronbach’s alpha = 0.59) and the MFQ-D (*n*_*ij*_ = 69, Cronbach’s alpha = 0.69) were lower than expected, which suggested that the selected items were not related to the same construct^j^.

**Figure 3:**
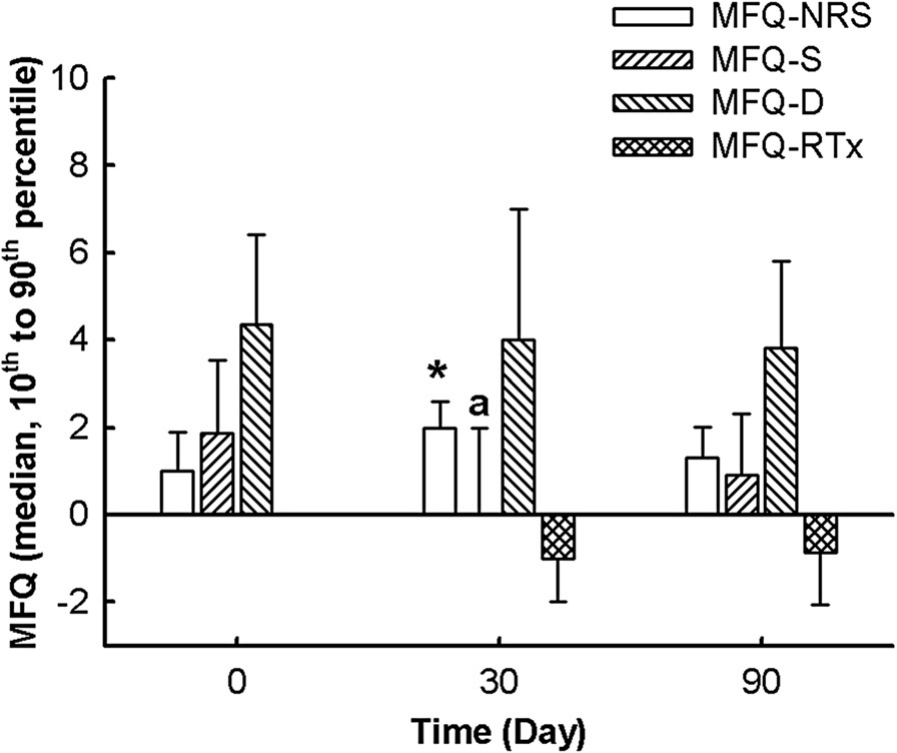
**Evolution of a multifactorial questionnaire (MFQ) in osteoarthritic dogs fed subsequentially with control and green-lipped mussel-enriched diets.** MFQ-NRS: MFQ-Numerical Rating Scale, MFQ-S: MFQ-Static, MFQ-D: MFQ-Dynamic, MFQ-RTx: MFQ-Response to Treatment. Data are represented as median, 25^th^ to 75^th^ percentile at day(D)0, D30, and D90. General linear mixed model for repeated measures: (**a**) was significant difference of Least Squares Means when compared with D0. Multinomial logistic regression for repeated measures: *significant odds ratio (D0 *vs* D30). Significance was reached at P-value lower than 0.017 with Bonferroni’s adjustment.

Figure 3 illustrates that the MFQ-NRS score was 3.0 times (OR) higher on D30 than on D0 (P_adj_ = 0.04), which indicates deterioration in the dogs’ condition. However, this score was unchanged from D0 to D90 (P_adj_ = 0.60) and from D30 to D90 (P_adj_ = 0.27). Moreover, the MFQ-S was lower on D30 (P_adj_ = 0.003) compared to on D0. No differences on D90 compared with D0 (P_adj_ = 0.21) or D30 (P_adj_ = 0.75) were noted for MFQ-S. Finally, the MFQ-D and the MFQ-RTx did not change over time (P > 0.05) (Figure 3). The divergent scoring of the MFQ subscales did not validate this standardized scale in the study.

## Discussion

The present study represents the continued interest in the conceptual and technical validities of multiple methods for pain assessment in canine OA. The PVF, CSOM, and MA demonstrated convergent results for the detection of diet-induced changes. The PVF exhibited the best technical validity, and the CSOM and MA demonstrated moderate technical validity. However, the CSOM evaluating the dogs’ day-to-day health status was found as related to the PVF and was primarily influenced by MA duration. The differences in the conceptual and technical validities suggested PVF, CSOM, and MA did not provide the same information on OA pain and physical function, but importantly they should be used and interpreted as a whole.

This study demonstrated that the PVF showed responsiveness to a GLM-enriched diet effect on canine OA. The study confirmed that the kinetic force platform gait analysis is a useful outcome because of the relative ease of data acquisition and technical validity [21,22,24,25,29-31]. Interestingly, age, sex and radiographically estimated OA severity were not affecting PVF, and the value of PVF representing the most affected limb was an accurate outcome measure for OA. But it might only partially represent the consequences of the entire disease on the physical function in these selected dogs, with regards to the absence of relationship between PVF and MA.

Clinical owner-oriented outcomes would be conceptually preferred, in general, because they are expected to reflect a dog’s quality of life. The questioning of the patient/owner about the quality of life has opened a wider panel of OA physical function and pain assessments. The CSOM is a subjective instrument that satisfied some psychometric qualities in this clinical trial. The owner-described behaviors in the CSOM were similar to previously described behaviors in canine studies of OA symptoms and pain [7,19]. These similarities suggest the face validity^k^ of the CSOM, which indicates that this measurement assesses the appropriate outcome [32]. Reliability testing revealed that the owner’s assessment was not variable over a one-week period. This is suggestive that CSOM scores were stable at the condition the dog’s status did not change between these two successive CSOM assessments (when facing a change, it was expected the owner to alert the investigators). With the conditions of the study, it was not possible to judge on a memory effect (the owner is influenced by the souvenir of the previous scoring) and the potential effect of a random error in CSOM owner assessment. Moreover, regression analyses demonstrated that both CSOM and PVF methods were related, supporting our second hypothesis. But, CSOM measurements were not strictly parallel to PVF (confirming our first hypothesis) as only the PVF detected a strict beneficial effect of the GLM diet. Divergent results between CSOM and PVF for the responsiveness analysis and the criterion validity analysis might be explained by the technical features of the CSOM. Indeed, the quantification scale of the CSOM is limited, likely decreasing its responsiveness. In addition, it was difficult to demonstrate a further decrease in the CSOM beyond D30 suggesting a possible floor effect. The sample size of 23 dogs was based on the power of the trial to detect a specified clinical benefit on the PVF. According to previous work done in similar conditions [25], a sample size of 20 dogs ensured a difference of 4.2%BW in the PVF consistent with an effect size of 0.9 could be detected assuming 80% power, a SD of 4.5 and a 5% significance threshold. This sample size might be too weak to fully support GLM diet efficacy using CSOM. This result suggests that the CSOM would decrease the likelihood of false clinical benefits. In contrast, PVF was responsive to GLM effect. Whereas PVF demonstrated significant change between D30 and D90, the primary endpoint CSOM has not demonstrated a statistically significant change for the same interval time. This could look as controversial for the validity of the methods. Therefore, we applied a correction for multiplicity of endpoints that adjusted the Type I error when a significant result was required for more than one but not all multiple primary outcomes after correction of multiplicity [33]. With the correction for multiplicity, PVF was significantly different between D30 and D90. This method provided stronger evidence to fully characterize the metrological feature of the methods when a small treatment effect is anticipated. In addition, the study suggests the two methods complementary relationship in pain assessment in which the CSOM assessed pain-induced changes in behavior and locomotion, and the PVF analyzed kinetic gait changes.

Previous studies have not indicated the details of the construction of the CSOM [19]. As previously noted, owners easily report on their dog’s activities, but the number (from 5 to 3 activities) and the nature of the selected activities in the CSOM differed between users [19,34]. This result suggests that OA has a variable impact on the daily life of dogs or that some owners missed behavioral changes that are caused by chronic pain [35]. In this study, an analysis of the influence of each activity revealed that the ranking of activity was neither valid nor informative. However, the categorization of the activities demonstrated significant behavioral changes. The owners detected higher limitations in activity in their dogs after exercise (Ctg_(2)_) or after a certain period of inactivity, including stiffness at night/after activity/in the morning (Ctg_(4)_). These behaviors are events that concur with suggestions of pain signs in previous studies. Hence, the selection of the same number of activities (we suggest 2 to 3 activities) that are worded by the owner but selected from the present categories would allow more precise and sensitive comparisons between dogs in future studies.

Our findings reported a significant difference in MA from P1 to P6 (Figure 2). However, the graph clearly suggests the possibility to detect more frequent differences while taking account individual variability of these data (*cf.* the actual difference with age on this limited sample) and required sample size to avoid Type II error. In addition, according to the small sample inference of the linear mixed model [36], our result needs to be verified on larger sample size. Despite limitation about the statistical inference, the present result about MA opens new insight in the field of pain evaluation for osteoarthritic dogs. Interestingly, MA did not significantly rely on PVF but to CSOM. These results emphasized that MA provided an aspect of spontaneous physical activity that was not detected by the conditioned gait evaluation, *i.e.* PVF. In addition, MA may be influenced by the proper ability of the dog to move freely and reflect its quality of life as defined by CSOM. Therefore, MA and CSOM are attractive outcome measures of pain and physical function in OA dogs because they are interrelated but different; one measurement is objective and the other is subjective.

The observed divergence in the results of the constitutive MFQ subscales (*e.g.*, MFQ-NRS and MFQ-S from D0 to D30), the lack of responsiveness (for MFQ-RTx and MFQ-D) and the weak internal consistency (particularly for MFQ-S) lead to poor technical validity and question the construct validity^l^ of this method. Although the CSOM and the MFQ were designed for clinical use to evaluate similar aspects of OA, the current results suggest that both pain scales partially reflect the same construct or that the MFQ did not satisfy adequate psychometric features. The standardized MFQ scale looked less potent to reflect the variability in limb impairment, activity limitations and other pain syndromes in canine OA.

The EDA measurement did not provide conclusive results in this clinical study. Invalidated outcome measures were observed in experimental conditions in rodents with a higher sympathetic tonus [37], but our own group has reported positive results with its use in the experimental (Pond-Nuki) canine OA model [31].

The GLM-enriched diet and the GLM extracts although not a reference treatment for OA were shown to exert a positive therapeutic effect on the clinical signs of dog OA [14,38]. Although a placebo-controlled trial would have been favored, ethical issues precluded a parallel comparison over 60 days between GLM- and placebo-treated dogs. Therefore, only a longitudinal one-way crossover design was included in the experimental design using the dogs as their own control. The anticipated effect of the GLM-diet was weak [14,38]. We did not know how much difference should be seen after two months of giving GLM. In addition, we observed slight improvement from D0 to D30 for CSOM, and MA seemed to be better during P2 (D16 to D29) two weeks after changing the home food for the control diet. Previous studies reported better owner-assessed pain in OA dogs with diet change [39,40]. Altogether, it is possible that the changes were not resulting from the GLM-diet but might be representing a trend over time caused by the diet changes [39]. Some might argue that PVF was not a good indicator of pain (or, at least claim that PVF is less sensitive than vertical impulse for hip dysplasia condition). As other critic in the present study, we could mention the fact that the other methods, *i.e.* MFQ and EDA, did not detect any treatment effect over the GLM-diet period. However, the sensitivity of MFQ and EDA was not previously investigated in other OA related-pain study in clinical condition, whereas the vertical ground reaction forces, such as PVF, have already proven their ability to detect change with NSAIDs treatment [22-25].

## Conclusions

This study investigated outcome measures that assessed signs in naturally occurring OA in dogs. The data from this study demonstrated that the limitation in activities (CSOM) and physical function, including limb impairment (PVF) and physical activity (MA), acted as complementary measures. Further developments on CSOM and MA would optimize their use in clinical trials.

## Methods

### Animals

The Institutional Animal Care and Use Committee approved the experimental protocol (Rech-1297) following the Canadian Council on Animal Care guidelines.

Thirty dogs were selected at the University teaching hospital of the Faculty of Veterinary Medicine (Université de Montréal, St-Hyacinthe, QC, Canada). The selection criteria were the following: 1) chronic and stable lameness, as reported by the owner; 2) no pregnancy and obesity; 3) weight greater than 20 kg (44 lb) and age greater than 12 months; 4) freedom from non-OA orthopedic, neurological and other abnormalities; 5) lack of orthopedic surgical treatments in the past year; and 6) no treatment with OA prescription-type diets, fatty acid supplements, continuous oral or injectable anti-inflammatory drugs that were prescribed by a veterinarian (including both steroid and NSAID), or polysulfated glycosaminoglycans therapy. Dogs that were receiving an oral nutraceutical or sporadic NSAID administration underwent a 4-week withdrawal period to establish eligibility.

When expressed as a percentage of their body weight (%BW), a PVF lower than 99.1% BW for forelimb lameness or lower than 62.2% BW for the lameness of one or more hindlimbs was required for inclusion, as previously performed [41]. An experienced veterinary surgeon performed an orthopedic exam and reviewed the digital radiographs of hips, stifles, and elbows for signs of OA. Physical examination, biochemical and hematological analyses (CBC, chemistry panel, and urine analysis) were performed on each dog. These were done to exclude non-healthy animals that presented non-OA orthopedic, neurological or other abnormalities. Dogs were removed from the study if either of the following conditions occurred: 1) the administration of a recognized treatment for OA, 2) medical problems or a sudden deterioration in orthopedic condition, 3) decision by the owner or 4) a change in housing style.

### Study design

All the dogs were fed a standard balanced diet of adult dry food (Dog Chow®, Nestlé Purina, St.-Louis, MO, USA) from D0 to D30 to standardize the food regimen for use as a placebo-control. This dog chow did not include any proclaimed active ingredients for the treatment or alleviation of OA. Subsequently, all the dogs were fed an experimental therapeutic food that was enriched in green-lipped mussel (GLM) from D31 to D90 (Mobility Support JS®, Medi-Cal/Royal Canin, Guelph, ON, Canada). The latter diet was chosen for its potent pain relief activity [14,42] without the side effects that are induced by the long-term administration of NSAIDs [43,44]. The packaging of both diets was identical. The pet owners and the investigators in the field had no knowledge of the food identity and in what order the diets were fed. The food composition and the daily food needs all met the recommendations of the Association of American Feed Control Officials for the maintenance of adult dogs [45]. The pet owners were instructed to transition their dogs to the assigned foods as following: a 14 transition day period at D0-D14 and at D30-D44.

The dogs remained at home throughout the study. The pain CSOM questionnaire and MA were recorded within the dog’s environment. The others methods were performed on D0, D30 and D90 at the University teaching hospital.

### Primary endpoints

#### Kinetic force platform gait analysis (PVF_BW_)

Data acquisition for the PVF was obtained for a trot at a constant velocity between 1.9 and 2.2 m/sec on a biomechanical force platform (Model OR6-6®; Advanced Medical Technology Inc., Watertown, MS, USA) coupled to software (Vetforce®; Sharon Software, Dewitt, MI, USA) as described previously [41]. The lowest PVF determined the most severely affected limb, which was selected for evaluation and used throughout the entire study. Five valid trials were recorded for the selected limb and averaged for further analyses. As recently reported by our group, PVF adjusted for individual BW changes (PVF_BW_) was used for the analyses [41].

##### Client-specific outcome measures (CSOM)

The CSOM scale is an owner-specific instrument to assess the influence of OA on the dog’s behavior [18]. A veterinarian assisted the owners at baseline, and the owners determined the five most difficult/OA-affected activities [Act_(i)_ for their dog. The evaluation of fewer than five activities was permitted. The owners ranked the activities by the level of importance from Act_(1)_ to Act_(5)_. Each activity was scored from 0 (no problem) to 4 (greatest difficulty) on a five-point Likert-rating scale. The median value of all the activity scores (CSOM_*Med*_ = Median [Act_(i)_) ranged from 0 (no problem/pain) to 4 (greatest difficulty/pain). Each owner completed the CSOM twice per week. The activities were also subjectively clustered into six categories [Ctg_(i)_ to provide a description of the activity limitations induced by OA: Ctg_(1)_ = Reduced mobility; Ctg_(2)_ = Reduced mobility after exercise; Ctg_(3)_ = Reduced ability to change posture; Ctg_(4)_ = Reduced ability to change posture after rest or in the morning; Ctg_(5)_ = Resistance to manipulations; and Ctg_(6)_ = Mood change. If more than one selected activity was included in the same category, a median value of these scored activities was used to score the category.

#### Electrodermal activity (EDA)

An EDA measurement was performed using the Pain Gauge system® (PHIS Inc., Dublin, OH, USA) to reflect sympathetic responses related to stress and pain. Both electrodes of the device were applied on a dry right hind paw for two seconds. The instrument displays a numeric level of skin conductance that ranged from 0.1 (no pain, no stress) to 9.9 (worst pain and stress). The measurements were performed in triplicate on D0, D30 and D90. The data were averaged at each time point for the statistical analyses.

### Exploratory endpoints

#### Motor activity (MA)

Accelerometer microchips (Actical® Mini Mitter, Bio-Lynx Scientific Equipment, Inc., Laval, QC, Canada) continuously recorded MA for 12 weeks on seven randomly selected dogs. These microchips are recent introductions in clinical studies, and we were interested in an *a priori* assessment of the technical validity of this tool in a limited sample. The sensor was placed on a neck collar. The epoch length of the count acquisition was set at 2 min. The intensity of the MA for each count was recorded and translated to numerical values (no unit). The data recorded at D0, D30 and D90 were externally excluded from analyses because they did not represent the true MA as the dogs were handled at the clinic. To reach the same interval of time for analysis, we excluded too D15, D45, D60 and D75. Therefore, data are represented in six consecutive periods (P_(i)_) of 14-day average total intensities. Three daily time periods (night from 20:00 to 07:00 h; morning from 07:02 to 13:00 h; and afternoon from 13:02 to 19:58 h) were established to stabilize data variability in further analyses. Moreover, the duration of MA was assessed over each 14-day period as the number of nonzero intensity counts.

#### Multifactorial pain questionnaire (MFQ)

An MFQ scale supporting OA disease-specific measures was designed, based on the content validity of previous reports [7-10]. Item selection and scale construction produced four subscales. The MFQ-Numerical Rating Scale (MFQ-NRS) is a five-point Likert scale (0–4) with 0 representing no pain and 4 the most excruciating pain perceived in dogs. The MFQ-Static (MFQ-S) scale was expected to measure the retrospective emotional functioning of the owner toward his/her OA-afflicted dog (Table 4). The MFQ-Dynamic (MFQ-D) scale measured prospective physical functioning (Table 5). Finally, the MFQ-Response to Treatment (MFQ-RTx) scale reported the satisfaction of treatment through a simple descriptive scale ranging from really better (−2); lightly better (−1); no change (0); lightly worse (1); to really worse (2).

**Table 4:** Presenting the static component of the multifactoral questionnaire (MFQ-S) used in the study

**From the following list, check any depressed or diminished parameter observed on your dog during the last month**	
Mood	
Amount of daily activity	
Willingness to play voluntarily	
Frequency of postures of a happy dog	
Appetite	
Sleep (disturbance)	
From the following list, check any change of attitude	
Social relationships (withdrawal) with humans	
Social relationships (withdrawal) with other dogs	
Vocalization (audible complaining)	
**Number of total observed parameter: Total score (/10)**	

**Table 5:** Presenting the dynamic component of the multifactorial questionnaire (MFQ-D) used in the study

**From the following list, check any parameter presently observed on your dog**	
Stiffness/difficulty to rise after rest	
Stiffness/difficulty moving at the end of the day (after activities)	
Lameness/difficulty to perform regular activity (walking, running…)	
Lameness/difficulty to perform brutal activity (jumping, stairs…)	
Pain when turning suddenly while walking	
Difficulty to sit or lay down	
Difficulty to rise from a lying position	
Difficulties to squat, urinate, or defecate	
**Number of total observed parameter: Total score (/8)**	

### Statistical analyses

#### Reliability

Variability in data outcome for the repeated measures was determined at each time-point, for each dog by calculating the mean coefficient of variation (CV), as previously performed [21]. The CV was calculated as the ratio of the standard deviation to the mean of the repeated measurements for the PVF (n = 5 at each time-point), the CSOM (n = 2 per week) and the EDA (n = 3 at each time point). For the CSOM, two values of the Act_(i)_ sum in the same week were considered as duplicates, and these values were used for the dispersion analysis. This analysis was compulsory for the CSOM_*Med*_ value distribution to approximate zero, which might mislead the CV. A mixed model analysis was used to compare changes in mean CV over time to estimate if the dispersion of the repeated measures was not different at each time point. The later was considered as good when time did not exert a significant effect in the model, finally, the internal consistency of the MFQ-S and the MFQ-D was assessed by the calculation of Cronbach's alpha. The threshold for good interrelatedness among items was set at > 0.7 [32].

#### Responsiveness

Our analyses examined the responsiveness of the PVF and MA methods using a mixed-model method for repeated measures with time as fixed effect and subject as random effect, and provided fixed effect estimates by restricted likelihood modeling. The homogeneity of the variance assumption using the probabilities of Levene’s test was provided for each time. Normality distribution was confirmed using the Shapiro-Wilk test. Log-transformation was performed to fit normality when required. For covariance structure selection, we used goodness-of-fit statistics to compare models with the same fixed effects but different covariance structure. Also, normality and homogeneity of the variance of the models’ residuals were checked to assess the model validity [46,47]. A multinomial logistic regression for repeated measures analyzed the CSOM_*Med*_, Act_(i)_, Ctg_(i)_, and MFQ subscales as discrete dependent variables. The Cochran-Mantel-Haenszel test was applied when the statistical model did not fit the assumptions of the multinomial logistic regression approach. A Poisson regression assessed EDA and the duration of activity (*i.e.*, the number of active MA counts). We addressed missing data using pairwise deletion. When required, factors such as age at inclusion (plus or minus 6 years), sex (male or female), the anatomical location of the most affected limb (cranial or caudal leg), and the severity of the OA lesion based on the number of joints diagnosed by radiography, were tested in the statistical models. Another factor as the daily time periods (night, morning, afternoon) was also tested for MA. Only the significant factors were included in the final statistical models.

#### Criterion validities

The descriptive statistics of the Spearman Rank correlation coefficient and their 95% interval confidence at the CSOM, EDA, MA endpoints were summarized by referencing their values to the PVF_BW_ at D0, D30 and D90 to assess concurrent validity^h^. The estimated 95% confidence limits represented the 25^th^ and 975^th^ rank-ordered values from the 1000 bootstrap resampling of 23 dogs [48]. For predictive validity^i^, we first described the magnitude of the systematic differences of the same outcomes between D0 and D30 and D30 and D90. Hence, the inferential statistics using regression analyses were performed to assess the relationship of the PVF_BW_ to the CSOM, the EDA and MA at the three time points. A regression analysis of the CSOM and MA was also performed on the continuous recordings of the seven dogs.

Statistical analyses were performed using the GLIMMIX and MIXED procedures in the SAS software (version 9.1, SAS Institute, Inc., Cary, NC). The α-level of significance was set at 5%. For the correction of the multiplicity of the clinical endpoints (PVF, CSOM, and EDA), the significant level of the statistical models was set at the α/(k) level, where an α of 5% is the original Type I error, and k is the number of primary endpoints to be performed [33]. As a result, the corrected Type I error probability was set at 0.05/3 = 0.017 for the Type 3 tests, which tested for the significance of each of the fixed effects specified in each model. When the statistical model result yielded significant main effect for time, Bonferroni’s adjustment was applied at the 5% α-threshold for *post-hoc* repeated measures of the same method (adjusted P-value cited as P_adj_). Descriptive statistics of the measured outcomes at each time are presented as the mean ± SEM and the median (25^th^ to 75^th^ rank-ordered values) for the ordinal data.

## Endnotes

^a^Conceptual validity is the *a priori* acceptation that the attribute (or scientific construct, *i.e.* canine osteoarthritic pain in our case) to be measured produces variations in the measurement outcomes (*i.e.* pain scales in the actual context). It refers to an ideal outcome that directly measures an important change in the patients' health status that is the result of the study intervention.

^b^Technical validity is the evidence base for technical properties of the methods including intra- and inter-reliability, and (a number of different forms of) validity.

^c^Psychometrics is the field of study consisting in a single psychological attribute (*i.e.* animal pain) measurement (metrology) with one or multiple items (*i.e.* behavioral characteristics as part of pain expression) and establishing the validation of the developed measurement method by referencing to its technical validity (also cited as psychometric features).

^d^Sensitivity is the ability to detect differences between subjects (or subjects between groups).

^e^Responsiveness is the ability to detect a change when a subject improves or deteriorates.

^f^Reliability concerns the random variability associated with the measurements. Reliability testing consists in determining that results are consistent across repeated measures over time and by different observers. Internal consistency is part of the assessment of the reliability in multi-item scales as it tests the homogeneity of the content of a composite scale.

^g^Criterion validities assess an instrument against a criterion measure known to be valid (also called “true value” or “gold standard”).

Criterion validity could be ^h^concurrent validity, which involves a parallel comparison of the new instrument against a well-established method, or ^i^predictive validity, which usually makes a prediction of future test results.

A ^j^construct is an explanatory variable, which is not directly observable and is constructed from a theoretical concept (see ^a^).

^k^Face validity refers to the degree of symptomatic resemblance between the developed instrument or outcome measurement and the intended topic or clinical condition.

^l^Construct validity refers to the degree to which a developed instrument measures the scientific construct that is designed to measure.

## Abbreviations

Act_(i)_: Activity “i”; BW: Body weight; Ctg_(i)_: Category “i”; CSOM: Client-specific outcome measures; GLM: Green-lipped mussel; NSAID: Non-steroidal anti-inflammatory drug; PVF_BW_: Body weight change-adjusted peak vertical force; MA: Motor activity.

## Competing interests

All authors declare no competing interest in this publication.

## Authors’ contributions

Conception and design: MM, BL, JPP, JMP, ET. Acquisition and interpretation of the data: SB, PR, MM, BL, JPP, ET. Collection and assembly of data: PR, SB, MM, BL, JPP, ET. Provision of study materials or patients: SB, MM, BL. Statistical expertise: PR, MG, ET. Drafting the article: PR, SB, ET. Critical revision of the article for important intellectual content: All authors. All authors read and approved the final manuscript. Acquisition of funding: ET, BL. Administrative, technical, or logistic support: PR, SB, MM, MG, ET. ET takes the responsibility for the integrity of the work as a whole.

## Authors’ information

ET is actual Chair of the Non-Human Species – Special Interest Group of the International Association for the Study of Pain ( http://www.iasp-pain.org/SIGs/NonHuman/), member of the Research and Education Committee of the International Veterinary Academy of Pain Management ( http://www.ivapm.org/) as well as founding member and secretary of the 4AVET association ( http://www.4avet.org).
